# Editorial: Precocious Gut Maturation: Environmental and Dietary Factors

**DOI:** 10.3389/fnut.2022.868722

**Published:** 2022-03-11

**Authors:** Björn Weström, Stefan G. Pierzynowski

**Affiliations:** ^1^Department of Biology, Lund University, Lund, Sweden; ^2^Department of Biology, Institute Rural Medicine, Lublin, Poland

**Keywords:** gut mucosa, dietary composition, maternal diet, microbial gut colonization, phytochemicals

Gut maturation during early life involves a series of irreversible, both structural and functional changes that culminate during weaning, when mothers milk is replaced with solid (complex) food. These changes affect the motility, secretion, absorption, and barrier properties of the gastrointestinal tract, nervous and hormonal regulation of gut function and the gut-associated immunity (1, 2). Despite being considered primarily as a pre-programmed process, regulating factors and mechanisms involved in gut maturation during ontogeny have not yet been fully elucidated.

Previous studies, using newborn animals as models for human infants, imply that gut maturation, culminating naturally at weaning or induced precociously by enteral provocation, for example by polyamines, lectins, or enzymes, is initiated by luminal cues affecting the intestinal epithelium. This activation results in proliferation and maturation of the mucosal layer, the underlying tissue layers, including the gut-associated lymphoid tissue, as well as the accessory gut organs, including the pancreas (3). Comparison of gut maturation processes in different species, such as rodents and ungulates, could assist in identifying pathways that are conserved across species, as well as those which are more species-specific. This will enable us to identify elements that are genetically hard-wired from those that are subjected to environmental regulation and thus may be manipulated in order to accelerate or slow down gut maturation.

The aim of the Research Topic was to highlight the progression of our knowledge in the regulation of gut maturation and how the process can be induced precociously or delayed through environmental and dietary intervention. Different aspects were addressed in the seven contributions included in the Research Topic, with five original research papers and two reviews.

Out of the seven contributions, four papers dealt with the effects of diet on precocious gut maturation.

The effect of early feeding on intestinal maturation in newly-hatched chicks was evaluated in the study by Reicher et al.. They showed that feeding chicks a standard starter diet early, upon hatch, compared to delaying feeding by 1 day, stimulated small intestinal epithelial proliferation and differentiation, which in turn expanded the digestive, absorptive and secretory cell populations during the post-hatch period of 10 days.

The effect of feeding formulas of differing protein content, as well as the addition of two growth promoters, leucine and hydroxy-methylbutyrate, on organ growth and intestinal function in pre-term pigs was studied by Buddington et al.. The authors found that the effect of protein and leucine on intestinal growth and function, i.e., brush-border carbohydrase activity, was greater for low-weight pigs compared to average-weight pigs. The study also revealed that the pre-term piglet could serve as a relevant model for the identification of formula components that promote gut growth and development in premature human infants.

Arevalo Sureda et al. studied the effects of feeding regimen, par-enteral vs. enteral feeding, and feeding diets on exocrine pancreas maturation in both pre-term and full-term piglets. It was found that pancreatic digestive enzymes showed different postnatal developmental patterns and responsiveness to enteral feeding. Trypsin increased with fetal age at birth, amylase increased with postnatal age, while lipase decreased with age. Colostrum inclusion to the diet increased amylase, but decreased trypsin, without any consistent effects on lipase. The low activities of the key enzymes, amylase and trypsin, in pre-term piglets indicate an immature pancreas and reduced digestive capacity. However, rapid enzyme increases appeared after birth following colostrum supplementation. The study confirmed that dietary manipulation also affects the gut accessory organs.

Mukonowenzou et al. reviewed recent findings on the impact of dietary medicinal plants and phytochemicals on gut maturation. They addressed different plant products that either directly interact with the gut mucosa, like phytohemagglutinin, or affect the gut microflora producing fermentation products, like SCFA, that indirectly have trophic effects and induce precocious functional maturation. They also reviewed the negative effects of plant products on gut function and maturation. The know-how and use of medicinal plants and phytochemicals, that in many cases are relatively cheap and readily available, being stimulatory of gut maturation may alleviate problems arising in live-stock animals or premature babies with an immature gut.

Wang et al. and Słupecka-Ziemilska et al. focused on the effects of maternal dietary composition during gestation and lactation, with either a high-fat diet or one supplemented with probiotics/symbiotics. The effects of feeding rat dams a high-fat diet during gestation and lactation on the intestinal development of their offspring at 3 weeks of age was investigated. The high-fat diet increased the pups body weight and adiposity and resulted in small intestinal hypertrophy. Epithelial brush border enzymes were affected, with decreased sucrase and increased aminopeptidase A activity observed. Intestinal contractility (*in vitro*) in response to cholinergic stimulation was also affected. The study revealed that maternal consumption of a high-fat diet negatively impacted the structural and functional maturation of the gut in her offspring and may predispose them to metabolic disorders later in life.

Wang et al. showed that supplementation of the sow's diet with probiotics (lactobacilli and saccharomyces) or symbiotics (xylo-oligosaccharides) during gestation and lactation improved the intestinal epithelial morphology of the weaned piglets. This diet affected the intestinal microflora balance with increased levels of bifidobacterial and clostridia observed, which were probably associated with the improved intestinal immunity. Thus, maternal interventions with probiotics or symbiotics are an alternative strategy to influence the intestinal microflora of the offspring, improving their gut maturation.

Related to this, the review by Socha-Banasiak et al. focused on one of the current hot scientific topics, that of fetal and neonatal microbial gut colonization and its impact on gut maturation in human infants. The authors bring up the controversial *in-utero* colonization and the effects of maternal microbiota colonization after birth on acceleration of infant gut development and its benefits on metabolic and immunological function.

The contributions of this Research Topic have highlighted some initiating cues and mechanisms involved in precocious gut maturation in mammals and birds, including many important livestock animals that in today's intensive production environment are commonly affected by gut-related complications due to immaturity. The knowledge could also be applied translationally to improve strategies for the prevention or treatment of gut-related complications arising in infants, especially those born pre-term, being underweight or formula-fed.

## Author Contributions

All authors contributed equally to manuscript writing, read, and approved the submitted version.

## Conflict of Interest

The authors declare that the research was conducted in the absence of any commercial or financial relationships that could be construed as a potential conflict of interest.

## Publisher's Note

All claims expressed in this article are solely those of the authors and do not necessarily represent those of their affiliated organizations, or those of the publisher, the editors and the reviewers. Any product that may be evaluated in this article, or claim that may be made by its manufacturer, is not guaranteed or endorsed by the publisher.

## References

[1] Walthall K Cappon GD Hurtt ME Zoetis T. Postnatal development of the gastrointestinal system: a species comparison. Birth Defects Res B Dev Reprod Toxicol. (2005) 74:132–56. 10.1002/bdrb.20040 15834902

[2] Rakoff-Nahoum S Kong Y Kleinstein SH Subramanian S Ahern PP Gordon JI. Analysis of gene–environment interactions in postnatal development of the mammalian intestine. Proc Nat Acad Sci USA. (2015) 112:1929–36. 10.1073/pnas.1424886112 25691701 PMC4343130

[3] Weström B Arévalo Sureda E Pierzynowska K Pierzynowski SG Pérez-Cano F-J. The immature gut barrier and its importance in establishing immunity in newborn mammals. Front Immunol. (2020) 11:1153. 10.3389/fimmu.2020.01153 32582216 PMC7296122

